# Measuring Plantar Flexor Voluntary Activation and Maximal Voluntary Contraction in a Portable, Seated Method: A Validity and Reliability Study

**DOI:** 10.3390/jfmk11010116

**Published:** 2026-03-10

**Authors:** Molly E. Coventry, Andrea B. Mosler, Paola T. Chivers, Brady D. Green, Ebonie K. Rio, Myles C. Murphy

**Affiliations:** 1 Nutrition and Health Innovation Research Institute, School of Medical and Health Sciences, Edith Cowan University, Joondalup, Perth, WA 6027, Australia; 2 La Trobe Sport and Exercise Medicine Research Centre, La Trobe University, Bundoora, Melbourne, VIC 3086, Australia; 3 Australian International Olympic Committee, La Trobe University, Bundoora, Melbourne, VIC 3086, Australia; 4 School of Medical and Health Sciences, Edith Cowan University, Joondalup, Perth, WA 6027, Australia; 5 Research Department, Child and Adolescent Health Service, Nedlands, Perth, WA 6009, Australia; 6 School of Health Sciences, The University of Notre Dame, Fremantle, WA 6160, Australia; 7 School of Allied Health, Human Services and Sport, La Trobe University, Bundoora, Melbourne, VIC 3086, Australia; 8 The Victorian Institute of Sport, Melbourne, VIC 3206, Australia; 9 Institute for Health Research, The University of Notre Dame, Fremantle, WA 6160, Australia

**Keywords:** calf, triceps surae, interpolated twitch technique

## Abstract

**Background**: Voluntary activation testing quantifies the ability of the motor nervous system to produce maximal force. Laboratory assessment of ankle plantar flexor voluntary activation is common, but field testing in practical settings is limited by equipment portability. We aimed to compare plantar flexor voluntary activation and maximal voluntary contraction (MVC) using a portable device with a standardised laboratory method and evaluate the test–retest reliability of the portable protocol. **Methods**: We performed a pseudo-randomised, crossover design. Participants completed two protocols: (1) portable force plate testing and (2) a laboratory-based isokinetic dynamometer. Voluntary activation was assessed using twitch interpolation via tibial nerve stimulation. Differences between protocols were analysed using generalised estimating equations. Reliability was assessed with the intraclass correlation coefficient (ICC), standard error of measurement (SEM), and coefficient of variation (CV). **Results**: Twenty healthy participants (8 females, 12 males; median age 28.5 years) were included. No difference between protocols was detected for voluntary activation (β = 0.6, *p* = 0.68). The portable protocol demonstrated good reliability (ICC = 0.85) and low measurement error (SEM = 2.56%, CV = 2.79%). **Conclusions**: We demonstrated that the portable protocol is a valid and reliable method for assessing plantar flexor voluntary activation. It is suitable for assessing within-subject changes over time and can reduce participant attendance burden for neurophysiological muscle testing.

## 1. Introduction

Voluntary activation assessment is a neurophysiological proxy for the percentage of muscle that can be recruited and activated by the motor nervous system during a maximal contraction [1]. The interpolated twitch technique is a common method for assessing voluntary activation; however, other methods such as the central activation ratio exist [2,3]. The interpolated twitch technique is typically considered the most accurate method to quantify voluntary activation, as it is more sensitive in detecting small increases in force from the stimulation [4]. It involves supramaximal nerve stimulation applied at the peak of a maximal muscle contraction, typically isometric, followed by a potentiated resting twitch at the same intensity [3]. A lower level of neural drive represents a larger superimposed twitch, indicating there is a larger proportion of motor units not being voluntarily activated by the nervous system during maximal voluntary muscle activation [1]. The amplitude of the superimposed twitch is not only influenced by the proportion of motor units voluntarily activated by the nervous system but also by the firing frequency [5].

The soleus and gastrocnemius are the primary ankle plantar flexors [6]. These muscles contribute to vertical and horizontal forces during walking and running [7,8]. They also contribute greatly to sport-specific movements such as acceleration and deceleration, hopping, jumping and change in direction [9,10,11]. Acute (e.g., calf muscle strain) and gradual (e.g., Achilles tendinopathy) onset injuries often occur during such activities [12]. The ability of the plantar flexors to meet these demands is dependent on determinants of force production, which includes maximal descending motor drive (i.e., voluntary activation). A systematic review with meta-analyses of ankle plantar flexor voluntary activation identified a normative value of ~91% (95% confidence interval: 90% to 93%) in healthy populations [13]. However, there are few published studies in pathological populations, and most studies have relatively small sample sizes [13]. Given the voluntary activation impairments observed in common pathological conditions such as osteoarthritis and anterior cruciate ligament injury [14,15,16], it is essential that reliable testing methods for assessing plantar flexor injuries are available.

Plantar flexor voluntary activation is commonly measured in a laboratory, with force quantified using an isokinetic dynamometer [13]. This approach may be a barrier to clinical testing in practical settings due to the high cost and lack of portability of equipment [17]. Furthermore, there are several methodological barriers to valid and accurate measurement of plantar flexor voluntary activation using these devices. The device used to collect plantar flexion force needs to sample at a rate sufficient to collect small increases in force from the stimulation. Commonly used devices such as isokinetic dynamometers typically have a sampling rate of 100–1000 Hz, unless combined with external hardware/software [18]. Low sampling rates potentially underestimate or do not detect the superimposed twitch (which only lasts between 100–200 ms) [19]. Additionally, the measurement error for isometric plantar flexion force in measurement devices is often higher than the superimposed and resting twitch force. The 6.6% standard error of measurement (~8 Nm at a plantar flexion torque of 120 Nm) commonly found with these measurement methods is comparable or greater than typical twitch sizes (~1–15 Nm) [20,21]. This measurement error may be attributed to the high system compliance within the attachments and components [20]. Compliance may reduce the size of the superimposed twitch and subsequently overestimate the voluntary activation level [22]. A portable testing protocol using force plates and a strap reducing compliance is a reliable method of assessing plantar flexor muscle strength [23]. Adapting this protocol for voluntary activation, which should theoretically overcome the methodological concerns related to voluntary activation testing in an isokinetic dynamometer, may improve accessibility while still providing valid and reliable data with low measurement error for researchers and practitioners.

Our study aimed to investigate if there is any detectable difference between a portable and a standardised laboratory testing method for assessing ankle plantar flexor voluntary activation and muscle strength (i.e., maximum voluntary contraction). We also evaluated the test–retest reliability of the portable plantar flexion voluntary activation testing protocol. We hypothesise that there will be no detectible difference between the protocols for voluntary activation and muscle strength and that the portable voluntary activation protocol will have good test–retest reliability.

## 2. Materials and Methods

### 2.1. Study Design

We performed a pseudo-randomised, crossover experimental study design between June 2025 and August 2025.

### 2.2. Ethical Considerations

This research received ethical approval from the Edith Cowan University Human Research Ethics Committee on the 6 March 2025 (2024-06061-MURPHY). The research was performed in accordance with the Declaration of Helsinki. All participants provided informed electronic consent (Appendix A).

### 2.3. Reporting Guidelines

This study was informed by, and reported in accordance with, the Strengthening the Reporting of Observational Studies in Epidemiology (STROBE) statement [24].

### 2.4. Participants

We included a convenience sample of participants aged between 18 and 50 years old, who subjectively rated themselves as recreationally active and were free of lower-limb musculoskeletal pain and injury. We excluded people with a prior history of significant lower limb injury, pathology and/or surgery that resulted in time loss from physical activity (e.g., calf muscle strain injury, Achilles tendinopathy, knee ligament rupture). We also excluded people with any neurological condition limiting lower-limb muscular strength or if they had contraindications for lower-limb nerve stimulation (Appendix B).

### 2.5. Setting

Participant-reported survey data were collected online via Qualtrics <48 h prior to the first testing session (Qualtrics, Provo, UT, USA). All physical assessments were performed in a dedicated neurophysiology laboratory at Edith Cowan University. Two separate protocols were performed by a single researcher (MEC) experienced in neurophysiological assessments [13,15]. Protocol one was the assessment of ankle plantar flexor voluntary activation using a portable testing method. Protocol two was the assessment of ankle plantar flexor voluntary activation using a standard laboratory method. Participants completed two sessions of protocol one and one session of protocol two. The three testing sessions per participant each took no longer than 45 min, with a wash out period of ≥48 h between sessions. The order of protocol 1 and 2 was randomised, with the second reliability session always being performed last.

#### 2.5.1. Protocol One: Portable Testing Method

**Figure 1:**
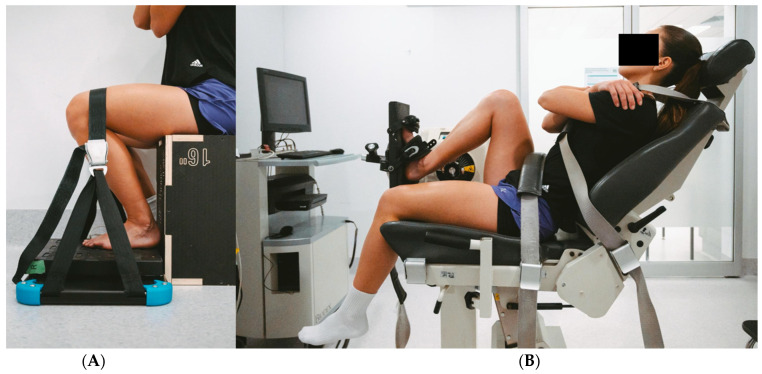
(**A**) Protocol one: portable testing method, (**B**) protocol two: laboratory testing method.

Participants were seated on an adjustable wooden box with hips flexed to 90 degrees and thigh parallel to the floor. The participant’s bare foot was placed on a force plate (PASCO PS-2141, PASCO scientific, Roseville, CA, USA) secured within the FysioMeter C-Station hardware (FysioMeter ApS, Aalborg, Denmark). The middle of the first and second metatarsophalangeal joints were positioned over the centre of the force plate. The ankle joint was placed into the participant’s maximal dorsiflexion and secured with a strap from the C-Station over the knee/distal thigh to limit limb movement (Figure 1A). The knee angle was set to position the participant in their maximal dorsiflexion range and standardised across testing sessions. The strap was tensioned maximally so minimal ankle movement occurred during strength testing and was periodically checked throughout the session to ensure it remained secure. Participants hip, knee and ankle joint ranges were measured with a 10″ 360° Liberty goniometer (Liberty Health Products, Melbourne, VIC, Australia) (hip and knee) and 6″ 360° 66fit goniometer (66fit Ltd., Spalding, Lincolnshire, United Kingdom (ankle) and recorded. Participants braced their upper body with their arms across their chest.

#### 2.5.2. Protocol Two: Laboratory Testing Method

Participants were seated in an isokinetic dynamometer (Biodex System 4, Biodex Medical Systems, Shirley, New York, NY, USA). The participants’ hip, knee and ankle joints were positioned with a gonimometer to match the participant’s position in the first protocol. If participants were randomised to perform protocol two first, they were set up in protocol one to measure joint position to allow for matching. The participants were secured to the dynamometer with straps over the shoulders and waist, and their feet were secured to the foot plate with two straps and foam padding for comfort (Figure 1B). Participants braced their upper body with their arms across their chest.

### 2.6. Variables

#### 2.6.1. Participants Characteristics

Age (years), sex recorded at birth (female; male; intersex, prefer not to say), ethnicity, body mass (kg), height (cm) and physical activity were self-reported. Physical activity was quantified using the World Health Organisation Global Physical Activity Questionnaire [25]. Participants reported minutes of moderate and vigorous activity per day within a typical week, and the final metabolic equivalent of task (MET) score was calculated as minutes of activity performed multiplied by MET score for activity multiplied by days of activity performed per week.

#### 2.6.2. Maximal Voluntary Contraction

Three warm-up trials at 25%, 50% and 75% of maximal effort were performed, and participants were offered further warm-up trials if required. Participants then performed three plantar flexion maximal voluntary contractions (MVCs) lasting 3 s per trial, with a minimum of a 60 s rest in the testing position. Participants were cued to “push directly through the ball of their foot”. The technique was visually monitored for contribution from the knee or hip extensors, and trials were repeated if required (e.g., due to technique error). Force (N) was recorded using a force plate (Pasco PS-2141, PASCO scientific, Roseville, CA, USA) or isokinetic dynamometer (Biodex System 4, Biodex Medical Systems, Shirley, New York, NY, USA), depending on the protocol. Real-time force data was displayed on a screen in front of the participant; however, they were blinded to their final MVC value from each session. The assessor was unable to be blinded between sessions. Both devices sampled at 2 kHz. Data were collected and recorded by Capstone (PASCO scientific, Roseville, CA, USA) or LabChart (ADInstruments NZ Limited, Dunedin, New Zealand) depending on the protocol. The maximal value of three trials was recorded in newtons (N).

#### 2.6.3. Ankle Plantar Flexor and Dorsiflexor Muscle Activity

Gastrocnemius (both medial and lateral heads) and soleus muscle activity was recorded via electromyography (EMG) during contraction and in response to the electrical stimulation (M-wave). The muscle activity and M-wave responses of the tibialis anterior were recorded to monitor antagonist muscles activation to refine the set-up protocol. Surface electrode (Kendall ECG electrodes) placement was informed by the surface electromyography for the non-invasive assessment of muscles (SENIAM) guidelines [26]. EMG signals were amplified and band-pass-filtered at 20–1000 Hz (BioAmp EMG System, ADInstruments NZ Limited, Dunedin, New Zealand) with a sampling rate of 2 kHz. Analogue signals were digitised and stored using LabChart (ADInstruments NZ Limited, Dunedin, New Zealand).

#### 2.6.4. Voluntary Activation

Voluntary activation was assessed using the interpolated twitch technique. The tibial nerve was stimulated in the popliteal fossa with a single 200 µs square-wave pulse delivered at 400 V via a constant-current stimulator (Digitimer DS7A, Digitimer Ltd., Hertforshire, UK). Single-pulse stimulation was selected as it is more comfortable for the participant and there is no significant difference in ankle plantar flexor voluntary activation levels when a single or double pulse is used [27]. The placement of the cathode (negative) was determined by the location eliciting the highest level of soleus EMG activity and plantar flexion force at rest, with minimal activation of the tibialis anterior. A hand-held probe (ADInstruments, NZ Limited, Dunedin, New Zealand) was used to determine the optimal location of cathode in the popliteal fossa before an adhesive electrode (Kendall ECG electrodes) was applied to the skin over the optimal location. The anode (positive) was placed over the medial tibial condyle. Single stimuli were increased in a stepwise fashion until no further increases in twitch amplitude and force were detected. Stimulation intensity for voluntary activation was set at 120% of the intensity required to evoke a maximum resting twitch in the plantar flexor muscles.

To perform the interpolated twitch technique, participants were instructed to perform an MVC while the assessor (MEC) provided strong verbal encouragement. When the force reached the participant’s maximum, the electrical stimulus was delivered manually by the assessor. The participant was instructed to relax completely, and once the EMG and force trace indicated the muscle was at rest, a second potentiated resting stimulus was delivered. A minimum of five voluntary activation trials were conducted with a 60 s rest between each effort. A trial was considered valid if the stimulation during the contraction occurred at their highest MVC for the trial. The valid trial with the highest pre-stimulation MVC was then selected. Voluntary activation (%) was calculated as:

$$Voluntary\;activation\left(\%\right)=\left(1-\frac{superimposed\;twitch\;amplitude}{resting\;twitch\;amplitude}\right)\times 100$$

Voluntary activation was calculated after completion of all testing sessions; therefore, both participants and the assessor were blinded to the voluntary activation results between sessions.

#### 2.6.5. Ankle Pain

Due to the potential for pain inhibition, the presence of any ankle pain in the testing position was recorded [28]. Ankle pain was rated by participants using an 11-point numerical rating scale, with 0 indicating no pain and 10 indicating the worst pain imaginable. Participants were asked to rate the pain in and around their ankle joint following the MVC and voluntary activation assessments.

### 2.7. Statistical Analysis

All statistical analyses were performed in the R language and environment for statistical computing (version 4.4.3). Age, sex, height, weight and physical activity were described using count, percentage, mean, standard deviation, median, interquartile range (IQR, 25th–75th quartiles), minimum (min), and maximum (max) where appropriate and based on normality testing. Normality of the data was assessed with the Sharpiro–Wilk test. Statistical significance was set at *p* < 0.05.

#### 2.7.1. Validity

Generalised estimating equations were used to evaluate differences in the dependent variable (model one: voluntary activation, model two: MVC). The protocol (laboratory/portable) and ankle pain (0–10) were included as a covariate in both models, with MVC included as a covariate in model one. The potential association of other independent variables to either voluntary activation or muscle strength were explored with violin plots and supplementary models. Model fit was assessed using Akaike Information Criterion (AIC), and residuals, multicollinearity, sensitivity analyses, and convergence were evaluated visually. Beta coefficients (β) and 95% confidence intervals (CIs) were reported.

Bland–Altman plots (systematic bias and 95% limits of agreement) were used to assess systematic bias between the laboratory and portable protocols. Model assumptions (normality, homogeneity of variance and proportional bias) were also assessed.

#### 2.7.2. Test–Retest Reliability

An intraclass coefficient (ICC), the standard error of measurement (SEM) and a coefficient of variation (CV) with 95% CIs were used to calculate the reliability of protocol one (i.e., voluntary activation between session 1 and 2). Reliability was rated in accordance with the recommendations by Koo and Li [29]. ICC estimates and 95% CIs were calculated based on a two-way mixed-effects model for absolute agreement using k = 5, as voluntary activation was determined from the best of multiple trials. ICC estimates less than 0.5 indicated poor reliability, those between 0.5 and 0.75 indicated moderate reliability, those between 0.75 and 0.9 indicated good reliability and values greater than 0.9 indicated excellent reliability [29].

## 3. Results

### 3.1. Participant Characteristics

We included 20 participants (8 female and 12 males), with demographics by sex presented in Table 1. Participants were a median [IQR, range] age of 28.5 years old [IQR = 26.0 to 30.5, range = 20 to 40], had body mass of 77.0 kg [IQR = 66.5 to 81.0, range = 53 to 109], and had height of 179.0 cm [IQR = 169.8 to 183.3, range = 160 to 196]. Participants identified as White (80%, 16/20), Asian (10%, 2/20) or Hispanic/Latino (10%, 2/20). Participant physical activity levels were a median [IQR, range] of 3600.0 METs/weeks [IQR = 2640.0 to 5340.0, range = 600 to 31,360]. The median [IQR, range] for knee position was 122.5° [IQR = 120 to 125, range = 155 to 130] and for ankle position was 10° of dorsiflexion [IQR = 10 to 15, range = 0 to 15]. The median [IQR, range] stimulation intensity used was 204 mA [IQR = 169 to 216, range = 120 to 300].

### 3.2. Ankle Plantar Flexor Voluntary Activation

#### 3.2.1. Validity

**Figure 2:**
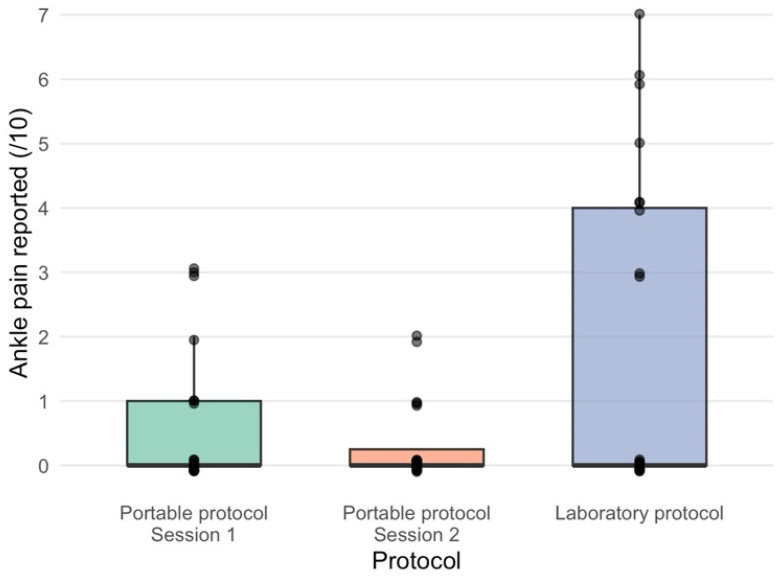
Subjective ankle pain (/10) reported during the portable protocol (session 1 and session 2) and the laboratory protocol. Boxplots display the median (central line) and interquartile range (box), with whiskers indicating the range of observed values.

The median [IQR, range] ankle plantar flexor voluntary activation when assessed with the portable protocol was 93.1% [IQR = 87.0 to 95.3, range = 72.9 to 98.5]. When assessed with the laboratory protocol, it was 93.5% [IQR = 81.9 to 94.5, range = 68.2 to 99.8] (Appendix C). The mean (SD) ankle plantar flexor MVC when assessed with the portable protocol was 996.9 N (285.2), and when assessed with the laboratory protocol, it was 844.0 N (272.4) (Appendix C). Participants reported a median [IQR, range] ankle pain during testing of 0/10 [IQR = 0 to 1, range = 0 to 3] in the portable protocol and 0/10 [IQR = 0 to 4, range = 0 to 7] in the laboratory protocol (Appendix C, Figure 2).

No difference was detected between the portable and laboratory protocols for plantar flexor voluntary activation (β = 0.6, 95%CI = −2.1 to 3.3, *p* = 0.676, Table 2) or MVC (β = −175.8, 95%CI = −365.5 to 14.0, *p* = 0.069, Table 3).

**Figure 3:**
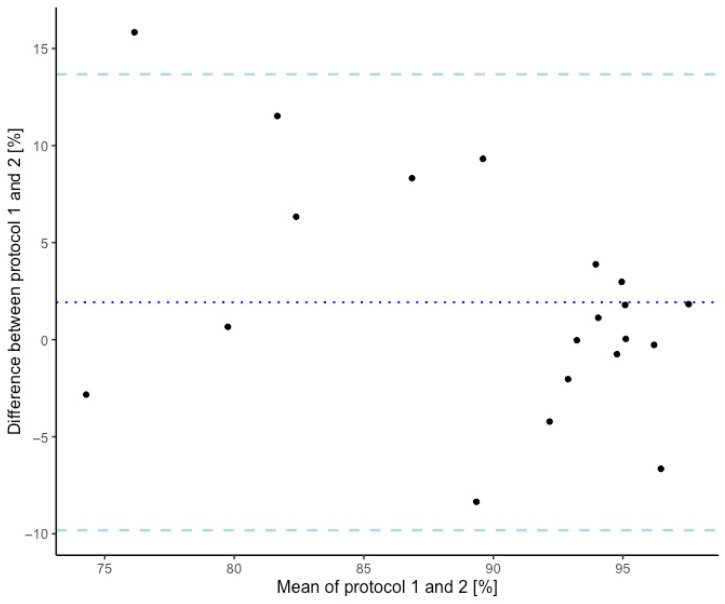
Bland–Altman plot of mean differences in ankle plantar flexor voluntary activation between the portable and laboratory protocols. Legend: dotted line = mean bias; dashed lines = 95% limits of agreement; protocol 1 = portable testing method; protocol 2 = laboratory testing method.

There was no proportional bias detected for voluntary activation (*p* = 0.059). All supplementary models are included within Appendix D. Bland–Altman plots showed a mean systematic bias of 1.93% (*p* = 0.167), indicating that the portable method may produce higher voluntary activation compared to the laboratory method. The lower and upper limits of agreement were −9.82% and 13.6% (Figure 3).

#### 3.2.2. Test–Retest Reliability

The median [IQR, range] ankle plantar flexor voluntary activation with the portable protocol for session one was 93.17% [IQR = 87.0 to 95.3, range = 72.9 to 98.4], and for session two, it was 94.4% [IQR = 90.6 to 97.3, range = 82.9 to 99.9] (Appendix C). The mean (SD) ankle plantar flexion MVC when assessed with the portable protocol for session one was 996.9 N (285.2), and for session two, it was 1082.8 N (228.11) (Appendix C).

Ankle plantar flexor voluntary activation assessed with the portable protocol (protocol 1) demonstrated good test–retest reliability (ICC = 0.85, 95%CI = 0.59 to 0.94). The SEM was 2.56% (95%CI = 2.02 to 3.27) and CV was 2.79% (95%CI = 2.15 to 3.58).

## 4. Discussion

This study was the first to measure the validity and reliability of a portable testing protocol to assess ankle plantar flexor voluntary activation. Our study did not detect a difference between a portable testing protocol using a force plate and a standard laboratory method using an isokinetic dynamometer in the assessment of plantar flexor voluntary activation and MVC, thus indicating that the portable protocol is a valid method of measurement. With good test–retest reliability and low measurement error, the portable protocol is well suited to assessing within-subject change over time.

Our study did not detect a difference between the portable and laboratory protocol for plantar flexor voluntary activation. We found a potential trend towards proportional bias whereby the portable protocol may overestimate voluntary activation at lower values; however, this was not statistically significant. The mean bias implies the portable protocol consistently produced two-percent-higher voluntary activation in comparison to the laboratory protocol. This reflects the higher level of plantar flexor force that the participants were able to produce in the portable protocol, which may have allowed for a higher voluntary activation level. The wide limits of agreement for systematic bias suggest that individual voluntary activation differences could be as large as 23% between the protocols, which may reduce the interchangeability of the protocols for absolute voluntary activation values.

No difference was detected between the protocols for plantar flexion MVC. The portable protocol appeared to record plantar flexion force values approximately 18% higher than those recorded in the laboratory protocol. Although the observed difference did not meet the threshold for statistical significance, the effect size exceeds the measurement error of isometric plantar flexion testing and warrants further consideration [21]. One potential reason the force may be higher in the portable protocol is there may be lower compliance/increased stiffness of the portable frame in comparison to the isokinetic dynamometer. A stiffer frame will transmit force more efficiently, rather than allowing force to be absorbed through equipment deformation [30]. This key biomechanical difference between the protocols may limit the comparability of the results. Participant body position also differed between protocols; the more upright seated position used in the portable protocol may have felt more natural for producing plantar flexion and may have placed the muscle in a more favourable position for force generation. However, using maximal dorsiflexion as the testing position enabled consistent standardisation of the ankle joint angle between protocols and sessions. Participants also reported less ankle pain in the portable protocol in comparison to the laboratory method, likely due to the absence of a foot/ankle strap, which may also have contributed to higher force production. A previous study has assessed the concurrent validity of a portable, seated plantar flexor strength testing device with an isokinetic dynamometer and demonstrated a good level of agreement (r = 0.72, 95%CI = 0.52 to 0.84, *p* < 0.01) [23]. Our results support these previous findings that the portable method is reliable, as well as being a more accessible alternative to standard isokinetic dynamometry for assessing maximal plantar flexor strength. This approach is advantageous for research involving pathological populations (e.g., calf injuries, stroke) where laboratory attendance is a challenge for recruitment.

The portable protocol test–retest reliability findings suggest moderate-to-excellent reliability and low measurement error across testing sessions [29]. Our results are in alignment with previous studies which assessed the reliability of plantar flexor voluntary activation in healthy populations (ICC = 0.858 and 0.837) [31,32]. In contrast, our ICC was higher than reported by Todd and colleagues (2004), who tested voluntary activation in a similar seated set up, although the authors attribute this to small variability among subjects and reported the reliability of the superimposed and resting twitches to be good [33]. Compared to the sample size of these studies (n = 5, n = 14, n = 5), our results may be better powered to provide a true estimate of plantar flexion voluntary activation in our testing position [31,32,33]. In our study, the corresponding standard error of the mean was small (indicating low absolute measurement error).

Our findings confirm that the portable testing protocol produces a valid measure of ankle plantar flexor voluntary activation and supports the use of the portable protocol to assess within-subject changes in plantar flexor voluntary activation in research and clinical settings. The reduced system compliance, more accurate sampling rate abilities and the portability of this set up likely yield more accurate data and improve testing accessibility. Future research could utilise the portable ankle plantar flexor voluntary activation protocol to assess pathological populations (e.g., calf muscle strain injury) to identify if similar deficits in voluntary activation seen previously in the literature exist for these injuries.

### Limitations

Whilst this study was powered to detect significant effects, the overall sample size and lack of a priori calculation may mean we could be at risk of type-one error. One limitation of the method comparison was the difference in body orientation and hip joint angle between protocols, which may influence the ability to produce plantar flexion force. However, hip, knee, and ankle joint angles were matched as closely as possible, and plantar flexion was elicited using the same verbal instructions in each protocol. Another limitation of this study is that reliability for the laboratory protocol was not assessed. Consequently, while the reliability results for the portable protocol are encouraging, we cannot directly compare them to the laboratory protocol, which would have strengthened our findings. Our sample was not recruited with equity, diversity and inclusion targets [34]; however, our included participants appeared to have varied demographic characteristics. Finally, the generalised estimating equation models were designed to detect differences between the protocols, and we were not powered to assess equivalence.

## 5. Conclusions

Our study demonstrated that the portable force plate protocol is a valid and reliable way to assess plantar flexor voluntary activation in healthy, recreationally active individuals. No statistically significant differences were detected for plantar flexion voluntary activation or MVC; however, equivalence was not formally tested, and interchangeability for absolute values may be limited. The portable protocol also demonstrated good test–retest reliability and low measurement error.

## Figures and Tables

**Table 1:** Participant characteristics.

Variable	Female		Male		Total	
	Mean (SD)	Median [IQR]	Mean (SD)	Median [IQR]	Mean (SD)	Median [IQR]
Age ^a^ (years)	29.00 (6.91)	28.50 [24.75–33.00]	28.50 (3.68)	28.50 [26.75–29.25]	28.70 (5.05)	28.50 [26.00–30.50]
Body mass ^a^ (kg)	62.75 (6.34)	63.50 [57.75–67.50]	82.75 (9.98)	80.50 [79.75–86.25]	74.75 (13.17)	77.00 [66.50–81.00]
Height ^a^ (cm)	168.12 (4.79)	169.00 [166.00–170.50]	183.04 (6.13)	182.50 [181.00–186.25]	177.07 (9.30)	179.00 [169.75–183.25]
Physical activity ^a^ (METs/wk)	3459.00 (2426.91)	3396.00 [1530.00–4560.00]	6168.33 (8093.10)	3600.00 [2850.00–5640.00]	5084.60 (6476.45)	3600.00 [2640.00–5340.00]

^a^ Indicates data are not normally distributed (Shapiro–Wilk test, *p* > 0.05); SD = standard deviation; IQR = 25th–75th percentiles; kg = kilograms; cm = centimetre; METs/wk = metabolic equivalent of task per week.

**Table 2:** Generalised estimating equation model evaluating the validity of a portable method of assessing voluntary activation.

	Voluntary Activation (%)		
Predictors	Beta Estimate	95% CI	*p*
(Intercept)	74.83	68.05–81.62	<0.001 *
Laboratory protocol ^a^	0.57	−2.11–3.26	0.676
Ankle pain (/10)	−0.03	−0.94–0.87	0.941
Maximal voluntary contraction (N)	0.02	0.01–0.02	<0.001 *

^a^ In comparison to the portable protocol; * indicates statistical significance. Akaike Information Criterion = 269.518; % = percentage; CI = confidence interval, *p* = probability value; N = newtons.

**Table 3:** Generalised estimating equation model evaluating the validity of a portable method for assessing plantar flexion maximal voluntary contraction.

	Maximal Voluntary Contraction (N)		
Predictors	Beta Estimate	95% CI	*p*
(Intercept)	985.46	866.35–1104.57	<0.001 *
Laboratory protocol ^a^	−175.75	−365.46–13.96	0.069
Ankle pain (/10)	16.34	−25.91–58.58	0.448

^a^ In comparison to the portable protocol; * indicates statistical significance. Akaike Information Criterion = 571.2069; CI = confidence interval, N = newtons; *p* = probability value.

## Data Availability

The de-identified data presented in this study are available on request from the corresponding author due to ethical restrictions.

## Appendix A. Electronic Consent Form

	Yes	No
I agree to take part in this research project.		
I have read the Information Sheet provided which explained the procedures involved and of what is expected of me.		
Prior to my survey being submitted, I understand that I may withdraw from participating in the project without prejudice.		
I understand that all information provided by me is treated as confidential and will not be released by the researcher to a third party unless required to do so by law.		
I agree that any research data gathered for the study may be published provided my name or other identifying information is not disclosed.		
I understand that research data gathered may be used for future research but my name and other identifying information will be removed.		
I understand that a MRI scan will be performed to confirm my diagnosis (if I do not already have one) and that the report for this MRI will be accessible to the research team.		
Please sign below to confirm your responses to the previous questions. Date:		
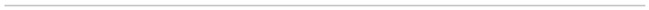

## Appendix B. Exclusion Criteria

Pregnancy
Neurological conditions/illness (including epilepsy/convulsion/seizure)
Vascular, traumatic, tumoral, infectious, or metabolic lesion of the brain
Previous or current implants in their body that may be triggered or heated by an electrical current (e.g., cardiac pacemaker, intracranial shunts, artificial cochlea, medication infusion device)
Any metal implanted in their head (e.g., surgical clips, staples, shrapnel)
Frequent or intense headaches
Ringing in the ears or hearing problems
Previous brain trauma or neurosurgical intervention to the brain or spinal cord
Serious medical complications (e.g., advanced pulmonary, cardiac, liver or kidney disease)
Currently taking neuropsychotropic drugs (e.g., antiepileptics, neuroleptics, benzodiazepines, antidepressants) or drugs with an effect on neuroplasticity (dopamine, fluoxetine or D-amphetamine, sodium or calcium channel blockers, NMDA receptor antagonists)

## Appendix C. Summary of Results

		**Female**			**Male**			**Total**		
**Protocol**	**Variable**	**Mean (SD)**	**Median [IQR]**	**Range (Min–Max)**	**Mean (SD)**	**Median [IQR]**	**Range (Min–Max)**	**Mean (SD)**	**Median [IQR]**	**Range (Min–Max)**
Portable protocol session 1	VA (%)	85.98 (7.43)	85.36 [83.07–91.55]	72.87–95.90	93.99 (3.01)	94.50 [92.87–96.00]	87.42–98.45	90.78 (6.47)	93.17 [86.96–95.32]	72.87–98.45
	MVC ^a^ (N)	726.86 (187.59)	769.25 [705.96–837.99]	293.93–881.54	1176.92 (172.48)	1136.00 [1087.41–1262.36]	955.98–1579.67	996.90 (285.24)	1040.73 [822.22–1139.95]	293.93–1579.67
	Pain (/10)	0.75 (1.39)	0.00 [0.00–0.75]	0.00–3.00	0.67 (0.98)	0.00 [0.00–1.00]	0.00–3.00	0.70 (1.13)	0.00 [0.00–1.00]	0.00–3.00
Portable protocol session 2	VA (%)	91.11 (6.85)	90.69 [84.56–97.55]	82.85–99.93	94.80 (3.30)	95.24 [93.52–97.24]	88.13–98.80	93.32 (5.20)	94.36 [90.58–97.25]	82.85–99.93
	MVC ^a^ (N)	876.49 (151.19)	899.14 [835.51–948.79]	556.67–1069.16	1220.31 (154.09)	1238.75 [1072.22–1335.18]	1015.14–1438.05	1082.79 (228.11)	1065.73 [932.84–1269.78]	556.67–1438.05
	Pain (/10)	0.25 (0.71)	0.00 [0.00–0.00]	0.00–2.00	0.42 (0.67)	0.00 [0.00–1.00]	0.00–2.00	0.35 (0.67)	0.00 [0.00–0.25]	0.00–2.00
Laboratory protocol	VA (%)	83.82 (10.47)	81.06 [78.34–92.39]	68.23–99.79	92.22 (5.94)	94.05 [93.41–95.10]	75.90–96.62	88.86 (8.86)	93.47 [81.88–94.48]	68.23–99.79
	MVC ^a^ (N)	662.30 (201.30)	618.38 [520.27–797.41]	403.85–1017.33	965.17 (249.81)	950.07 [735.45–1152.96]	640.27–1341.33	844.02 (272.45)	804.12 [655.19–989.17]	403.85–1341.33
	Pain (/10)	0.50 (1.41)	0.00 [0.00–0.00]	0.00–4.00	3.17 (2.62)	3.50 [0.00–5.25]	0.00–7.00	2.10 (2.55)	0.00 [0.00–4.00]	0.00–7.00
^a^ Indicates data are normally distributed (Shapiro–Wilk test, *p* < 0.05); SD = standard deviation; IQR = interquartile range 25th percentile to 75th percentile; min = minimum; max = maximum; VA = voluntary activation; % = percentage; MVC = maximal voluntary contraction; N = newtons.										

## Appendix D. Supplementary Models and Plots

Supplementary models with additional covariates indicated that age and physical activity were significant when included within the plantar flexion voluntary activation model and that sex and body mass were significant when included in the plantar flexion maximal voluntary contraction model; however, the primary variable remained non-significant.

Voluntary activation model including sex.

	**Voluntary Activation**		
**Predictors**	**Estimates**	**95% CI**	***p***
(Intercept)	77.68	70.57–84.79	<0.001 *
Laboratory method ^a^	0.16	−3.77–4.09	0.936
Ankle pain (/10)	−0.34	−1.03–0.35	0.341
Maximal voluntary contraction (N)	0.01	0.00–0.02	0.034 *
Sex [M]	4.65	−1.17–10.47	0.117
^a^ In comparison to the portable protocol; * indicates statistical significance; Akaike Information Criterion: 268.9883.			

Voluntary activation model including age.

	**Voluntary ^a^**		
**Predictors**	**Estimates**	**95% CI**	***p***
(Intercept)	87.11	75.29–98.94	<0.001 *
Laboratory method ^a^	1.39	−2.60–5.37	0.495
Ankle pain (/10)	−0.32	−1.12–0.48	0.435
Maximal voluntary contraction (N)	0.02	0.01–0.03	<0.001 *
Age (years)	−0.52	−1.01–−0.02	0.041 *
^a^ In comparison to the portable protocol; * indicates statistical significance; Akaike Information Criterion: 265.2356. Voluntary activation model including body mass.			

Voluntary activation model including physical activity.

	**Voluntary Activation**		
**Predictors**	**Estimates**	**95% CI**	***p***
(Intercept)	74.05	67.33–80.78	<0.001 *
Laboratory method ^a^	0.32	−3.61–4.25	0.872
Ankle pain (/10)	0.08	−0.82–0.98	0.866
Maximal voluntary contraction (N)	0.02	0.01–0.02	<0.001 *
Physical activity (METs/week)	0.00	0.00–0.00	<0.001 *
^a^ In comparison to the portable protocol; * indicates statistical significance; Akaike Information Criterion: 268.8282.			

Maximal voluntary contraction model including sex.

	**Maximal Voluntary Contraction**		
**Predictors**	**Estimates**	**95% CI**	***p***
(Intercept)	769.74	667.89–871.59	<0.001 *
Laboratory method ^a^	−129.01	−268.25–10.24	0.069
Ankle pain (/10)	−17.05	−52.64–18.55	0.348
Sex [M]	398.48	264.67–532.30	<0.001 *
^a^ In comparison to the portable protocol; * indicates statistical significance; Akaike Information Criterion: 546.5288.			

Maximal voluntary contraction model including age.

	**Maximal Voluntary Contraction**		
**Predictors**	**Estimates**	**95% CI**	***p***
(Intercept)	507.41	2.61–1012.20	0.049 *
Laboratory method ^a^	−186.53	−361.13–−11.92	0.036 *
Ankle pain (/10)	24.04	−14.12–62.19	0.217
Age (years)	16.47	−0.81–33.75	0.062
^a^ In comparison to the portable protocol; * indicates statistical significance; Akaike Information Criterion: 567.986.			

Maximal voluntary contraction model including body mass.

	**Maximal Voluntary Contraction**		
**Predictors**	**Estimates**	**95% CI**	***p***
(Intercept)	−205.24	−632.66–222.18	0.347
Laboratory condition ^a^	−112.48	−250.10–25.14	0.109
Ankle pain (/10)	−28.85	−65.04–7.33	0.118
Body mass (kg)	16.35	10.34–22.36	<0.001 *
^a^ In comparison to the portable protocol; * indicates statistical significance; Akaike Information Criterion: 543.8199.			

Maximal voluntary contraction model including physical activity.

	**Maximal Voluntary Contraction**		
**Predictors**	**Estimates**	**95% CI**	***p***
(Intercept)	961.81	828.78–1094.84	<0.001 *
Laboratory method ^a^	−178.17	−367.08–10.75	0.065
Ankle pain (/10)	18.07	−24.02–60.15	0.400
Physical activity (METs/week)	0.00	−0.00–0.01	0.067
^a^ In comparison to the portable protocol; * indicates statistical significance; Akaike Information Criterion: 571.2069.			
